# Exposure to Meta-Analysis During Graduate and Postgraduate Trainings Predicts the Good Knowledge and Use of Randomized Controlled Trials, Systematic Reviews, and Meta-Analyses Among Nigerian Physiotherapists

**DOI:** 10.7759/cureus.58251

**Published:** 2024-04-14

**Authors:** Overcomer T Binuyo, Omotola A Onigbinde, Sunday J Arogundade, Daniel C Akaeme, Bijad Alqahtani, Khalid M Alkhathami

**Affiliations:** 1 Department of Physiotherapy, Federal Medical Centre, Asaba, NGA; 2 Department of Wellness and Physiotherapy, OneHealth Medical Center, Ikeja, NGA; 3 Department of Physiotherapy, University of Medical Sciences Teaching Hospital, Ondo City, NGA; 4 Department of Health Rehabilitation Sciences, College of Applied Medical Sciences, Shaqra University, Shaqra, SAU

**Keywords:** evidence-based practice, physiotherapy education, meta-analysis, systematic review, randomized controlled trial (rct), physiotherapy

## Abstract

Background

Evidence-based practice (EBP) is essential for physiotherapy as an integral part of the multidisciplinary rehabilitation team. Randomized controlled trials (RCTs), systematic reviews, and meta-analyses are the gold standard in the hierarchy of evidence. However, the extent of knowledge, attitudes, and professional use of RCTs and meta-analyses among physiotherapists in Nigeria remains unclear. Therefore, this study aimed to describe and explore the predictors of Nigerian physiotherapists’ knowledge, attitudes, and professional behaviors toward RCTs, systematic reviews, and meta-analyses.

Methods

In this observational study, an electronic version of an adapted questionnaire assessing the knowledge, attitudes, and professional use of RCTs and meta-analyses was shared across electronic platforms of Nigerian physiotherapy professional organizations.

Results

We found good overall knowledge (76 {80.8%}) and attitude (83 {88.3%}) toward the use of RCTs, systematic reviews, and meta-analyses for evaluating health interventions. Exposure to meta-analysis during graduate and postgraduate training (odds ratio {OR}, 7.102; 95% CI, 1.680-30.021; p = 0.008) and the presence of a medical library at the workplace (OR, 0.264; 95% CI, 0.070-0.997; p = 0.049) were significant predictors of good knowledge of RCTs, systematic reviews, and meta-analyses. Self-rated (OR, 56.476; 95% CI, 1.356-2357.430; p = 0.034) and overall levels of knowledge (OR, 0.013; 95% CI, 0.000-0.371; p = 0.011) predicted the good use of RCTs, systematic reviews, and meta-analyses among respondents.

Discussion

To equip physiotherapy practitioners with the requisite skill in using RCTs, systematic reviews, and meta-analyses, graduate and postgraduate trainings should prioritize education on the use of RCTs, systematic reviews, and meta-analyses to inform clinical decisions and practice, while capable workplaces may set up medical libraries to ease access and enhance the use of RCTs, systematic reviews, and meta-analyses.

## Introduction

Evidence-based management (EBM) is central to the delivery of physiotherapy interventions because physiotherapy plays a critical role in patient recovery and rehabilitation [1]. EBM ensures the incorporation of the current best available evidence in literature with professional expertise in patient care. Twenty-first-century healthcare requires evidence-based interventions. With the growing local and global need for rehabilitation services [2,3], engaging in research to practice EBM is unavoidable for physiotherapists [4]. To practice evidence-based delivery, a health professional must recognize and define a problem, conduct efficient research to locate and critically appraise the best available evidence [5], and integrate this evidence with clinical expertise within the context of the patient.

Randomized controlled trials (RCTs), systematic reviews, and meta-analyses are considered gold standards in the hierarchy of evidence in literature [6]. RCTs enable the highest reduction of bias in the research of a cause-and-effect relationship between any two variables or measures. Usually, a target population, an outcome of interest, and interventions are selected. This study design ensures a random allocation of participants into groups without their prior knowledge and a blinding of researchers to the intervention being delivered [6]. However, results from RCTs are usually heterogeneous and not generalizable to every member of the same or different population [7], hence the need for systematic reviews and meta-analyses, which are aimed at pooling and summarizing all available evidence. Systematic reviews and meta-analyses involve a comprehensive and near-exhaustive literature search in databases, registries for unpublished articles [7], and grey literature to answer a research question. In writing a high-quality systematic review, the Preferred Reporting Items for Systematic Reviews and Meta-Analyses must be adhered to. Systematic reviews qualitatively summarize the results of a compendium of studies, and in the absence of statistical heterogeneity between included studies, a quantitative summary, the meta-analysis, is performed [7]. Arguably, many barriers, such as inadequate time, the lack of adequate training in research methodologies, the lack of support and funding, and the inadequate description of interventions in the available literature [8] militate against incorporating evidence with physiotherapy practice; nonetheless, interested practitioners still approach easily applicable secondary sources of evidence in health service delivery [9]. Conversely, the ability to engage in high-quality and intervention-based experimental research is paramount to physiotherapists. In Nigeria, the extent of the knowledge, attitudes, and professional use of RCTs and meta-analyses among physiotherapists remains underexplored; therefore, this study aimed to describe and explore the predictors of Nigerian physiotherapists’ knowledge, attitudes, and professional behaviors toward RCTs, systematic reviews, and meta-analyses.

## Materials and methods

Study design

This study was a cross-sectional study that used purposive sampling.

Subjects

The subjects were registered Nigerian physiotherapists who are members of the professional electronic platforms of the Medical Rehabilitation Therapists Board of Nigeria, the Association of Clinical and Academic Physiotherapists of Nigeria, and the Nigeria Society of Physiotherapy. Assuming an indefinite population of registered physiotherapists practicing in Nigeria, to obtain this study’s sample size, Cochran’s sample size formula for unknown population size, with a 95% reliability level, was used as follows: where n = sample size, p = adjusted population proportion (0.5), e = acceptable sampling error (0.05), and z = z value at reliability level (1.96 at 95% reliability level) [10].

Considering the 10% attrition rate to the nearest whole, the sample size for this study was 150.

Materials

An electronic version of an adapted questionnaire [5] was used to assess the knowledge, attitudes, and professional use of RCTs and meta-analyses among Nigerian physiotherapists across the electronic platforms of the professional bodies mentioned above between May and July 2023. The questionnaire had four sections: Section A comprised questions about sociodemographics, Section B comprised questions on professional characteristics, Section C comprised questions on the self-appraisal of knowledge about RCTs and meta-analyses, and Section D comprised questions on the knowledge, attitude, and professional use of RCTs and meta-analyses. The knowledge and attitude aspects of Section C obtained information using a three-point Likert scale, “Agree,” “Uncertain,” and “Disagree,” while the professional use aspect obtained information using a five-point Likert scale, “Never,” “Rarely,” “Sometimes,” “Often,” and “Very Often.”

Procedure

The overall scores in these subsections were determined by appending a mark of 1 for correctness and 0 for incorrectly answered questions, and aggregate scores were obtained. The respondents with scores greater than 50% had good knowledge and attitudes. This study was conducted in accordance with the principles of the Declaration of Helsinki. The survey instrument was accompanied by a cover letter to obtain written informed consent. Statistical analysis using descriptive statistics to summarize the data and logistic regression analysis with backward elimination to determine the factors predicting the respondents’ levels of knowledge, practice settings, and participation was performed using the Statistical Package for Social Sciences (SPSS) (version 20.0, IBM SPSS Statistics, Armonk, NY). Statistical significance was set at p < 0.05.

## Results

Physiotherapists’ sociodemographics and professional characteristics

Ninety-four physiotherapists responded to our survey resulting in a 66% response rate. Most respondents were males (62 {66%}) aged 26-30 years (47 {50%}). As shown in Table 1, among the respondents, 13 (14%) had between five and 10 years of practice (post internship), mostly in tertiary/teaching hospital practice settings (955 {58%}). The majority of the respondents had a bachelor’s degree as their highest qualification (84 {89%}), with no postgraduate training experience (64 {68%}). Among the respondents, 31 (33%) regularly dedicated time to continuing their medical education. Internet services and medical libraries in the workplace were accessible to 43 (46%) and 39 (42%), respectively. Table 1 shows the sociodemographic and professional characteristics of the respondents.

**Table 1:** Physiotherapists’ sociodemographic and professional characteristics RCTs, randomized controlled trials; PP, private practice; BMR, bachelor of  medical rehabilitation; DPT, doctor of physiotherapy

Variables	N	%
Age (in years)		
20-25	15	16.0
26-30	47	50.0
31-35	17	18.1
36-40	3	3.2
41-45	7	7.4
Over 45	5	5.3
Gender		
Female	32	34.0
Male	62	66.0
Religion		
Christianity	87	92.6
Islam	7	7.4
Years of practice (post internship)		
Less than five	64	68.1
5-10 years	13	13.8
10-15 years	11	11.7
15-20 years	2	2.1
Over 20 years	4	4.3
Practice setting		
Home service (PP)	7	7.4
Primary health facility	4	4.3
Private hospital	12	12.8
Private rehab facility	1	1.1
Secondary/general hospital	11	11.7
Specialist	3	3.2
Sports medicine center	1	1.1
Tertiary/teaching hospital	55	58.5
Highest Qualification		
BSc/BMR	84	89.4
DPT	1	1.1
Masters	8	8.5
PhD	1	1.1
Postgraduate (PG) training		
No PG training	64	68.1
Nonspecialized PG training	8	8.5
Specialized PG training	22	23.4
Dedication to continuing medical education (average)		
Monthly	13	13.8
Quarterly	10	10.6
Randomly	23	24.5
Regularly	31	33.0
Yearly	17	18.1
Internet at the workplace		
No	51	54.3
Yes	43	45.7
Medical library on the workplace		
No	55	58.5
Yes	39	41.5
Rate your level of knowledge of meta-analysis		
Inadequate	55	58.5
Sufficient	22	23.4
Good	14	14.9
Excellent	3	3.2
Do you need training/more training on RCTs and meta-analyses		
Yes	87	92.6
No	7	7.4

Knowledge of RCTs, systematic reviews, and meta-analyses

**Figure 1:**
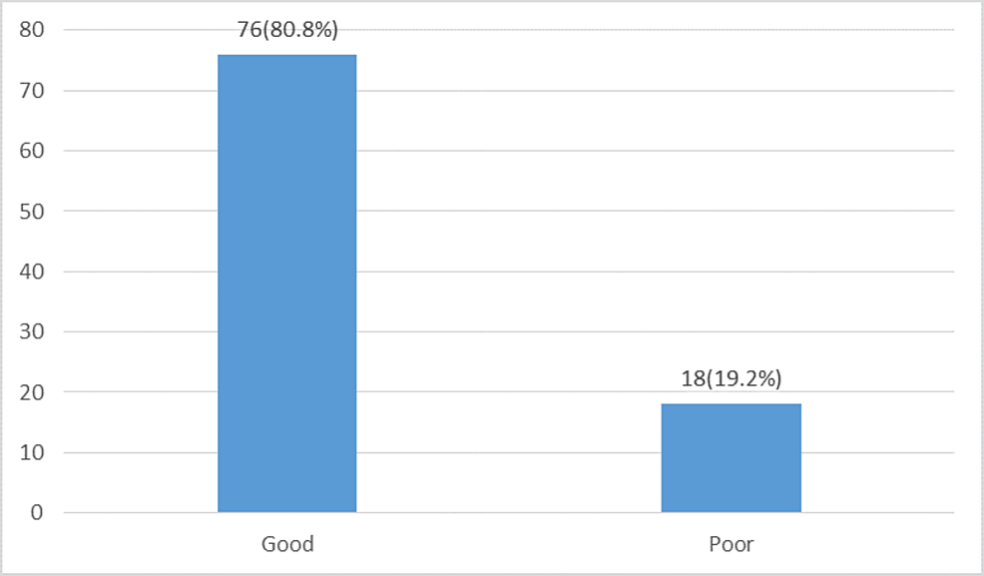
Physiotherapists’ overall level of knowledge

Table 2 summarizes the responding physiotherapists’ knowledge of RCTs, systematic reviews, and meta-analyses. The overall knowledge of the participants on RCTs and meta-analyses was good (76 {80.8%}) (Figure 1). However, only a little above average (49 {52.1%}) agreed that relative risk and odds ratio (OR) are measures used in RCTs and meta-analyses to quantify the effect of health interventions. Similarly, 55 (58.5%) rated their level of knowledge of meta-analyses as inadequate, and 87 (92.6%) affirmed that they would need training/more training on RCTs and meta-analyses (Table 1). Univariate analysis revealed a significant association between exposure to meta-analysis during graduate and postgraduate training (p = 0.002) and the level of knowledge in responding physiotherapists. Logistic regression analysis with backward elimination confirmed that exposure to meta-analysis during undergraduate and postgraduate training and the presence of a medical library at the workplace could significantly predict good knowledge (Model 1 in Table 3).

**Table 2:** Physiotherapists’ knowledge of RCTs, systematic reviews, and meta-analyses RCTs: randomized controlled trials

Items	Agree, n (%)	Uncertain, n (%)	Disagree, n (%)
RCTs are able to evaluate the efficacy of preventive and curative health interventions	60 (63.8)	33 (35.1)	1 (1.1)
Meta-analysis is useful to draw conclusions about the efficacy of health interventions	75 (79.8)	18 (19.1)	1 (1.1)
Meta-analysis combines the results of different individual studies with the purpose of integrating the findings	74 (78.7)	20 (21.3)	0 (0.0)
Relative risk and odds ratio are measures used in RCTs and meta-analyses to quantify the effect of health interventions	49 (52.1)	43 (45.7)	2 (2.1)

**Table 3:** Predictors of the knowledge and good use of RCTs, systematic reviews, and meta-analyses RCTs, randomized controlled trials; OR, odds ratio

Variable	OR	95% CI	P-value
Model 1: knowledge about RCTs, systematic reviews, and meta-analyses			
Exposure to systematic reviews and meta-analyses during graduate and postgraduate training (no = 0; yes = 1)	7.102	1.680-30.021	0.008
Medical library on the workplace (no = 0; yes = 1)	0.264	0.070-0.997	0.049
Model 2: appropriate use of RCTs/systematic reviews/meta-analyses			
Rate your level of knowledge (1 = inadequate; 2 = sufficient)	56.476	1.356-2357.450	0.034
Knowledge (poor = 0; good = 1)	0.013	0.000-3.71	0.011

Attitudes toward RCTs, systematic reviews, and meta-analyses

**Figure 2:**
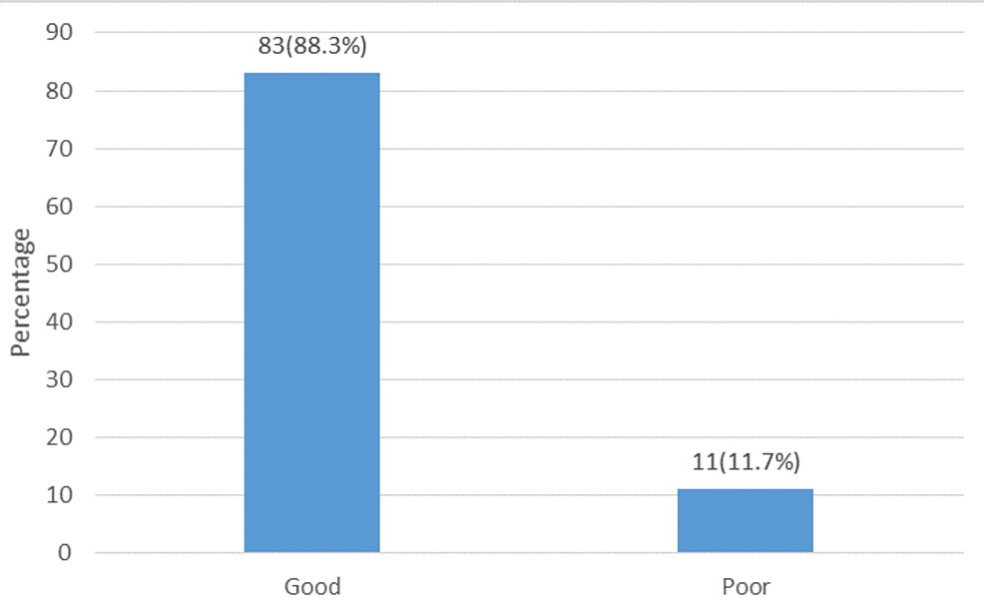
Physiotherapists’ overall attitude toward the use of RCTs, systematic reviews, and meta-analyses RCTs: randomized controlled trials

Table 4 summarizes the attitudes of responding physiotherapists toward using RCTs, systematic reviews, and meta-analyses as methods for evaluating the efficacy of health interventions. Their overall attitude was good (83 {88%}) (Figure 2). While the majority of the respondents agreed to the queries in the attitude section, a minority (39 {41%} and 45 {48%}) agreed that only health interventions with proven efficacy should be freely administered to the population and decisions in clinical practice cannot be based on the results of meta-analyses but rather on individual patient needs, respectively. Univariate analysis revealed a significant association between exposure to meta-analysis during graduate and postgraduate training (p < 0.001), the level of knowledge (p < 0.001), and attitudes toward the use of RCTs and meta-analyses by physiotherapists. However, multiple regression analyses revealed that none of these factors were a predictor of a good attitude toward RCTs, systematic reviews, and meta-analyses.

**Table 4:** Physiotherapists’ attitude toward the use of RCTs, systematic reviews, and meta-analyses RCTs: randomized controlled trials

Variables	Agree, n (%)	Uncertain, n (%)	Disagree, n (%)
Systematic reviews and meta-analyses contributed significantly to knowledge about the prevention and cure of diseases	71 (75.5)	23 (24.5)	0 (0.0)
Only health interventions with proven efficacy should be free	39 (41.5)	41 (43.6)	14 (14.9)
The application of results of RCTs, systematic reviews, and meta-analyses improves the health status of patients	74 (78.7)	19 (20.2)	1 (1.1)
Systematic reviews and meta-analyses are useful tools to help physiotherapists to select effective health interventions	84 (89.4)	10 (10.6)	0 (0.0)
Clinical practice requires efficacy evaluations of health interventions carried out through meta-analyses	76 (80.9)	17 (18.0)	1 (1.1)
Many decisions in clinical practice cannot be based on the results of RCTs, systematic reviews, and meta-analyses but rather on the individual patient needs	45 (47.9)	38 (40.4)	11 (11.7)

Predictors of the professional use of RCTs, systematic reviews, and meta-analyses

Our analysis revealed that the self-rated level of knowledge of meta-analyses (OR, 56.476; CI, 1.680-30.021) and the overall level of knowledge (OR, 0.011; CI, 0.000-0.371) (Table 3) were significant predictors of the professional use of RCTs, systematic reviews, and meta-analyses by the responding physiotherapists.

## Discussion

Our study showed that the majority (80.9%) of the responding physiotherapists had good knowledge of RCTs, systematic reviews, and meta-analyses. Nevertheless, only a little above average of the respondents knew that relative risk and odds ratio are measures used in RCTs, systematic reviews, and meta-analyses to quantify the effect of health interventions. In a similar study that explored Italian physiotherapists, more than 85% were familiar with the principles of evidence-based practice (EBP), although physiotherapists overrated their knowledge of EBP [11]. Familiarity can create an illusion of knowledge in conducting and using these research methodologies without the required expertise. This may limit the inspiration for further training and usage of RCTs, systematic reviews, and meta-analyses among physiotherapists. In this study, the predicting factors for good knowledge about RCTs, systematic reviews, and meta-analyses were exposure to meta-analysis during graduate and postgraduate training and the presence of a medical library at the workplace. Evidence shows that across several disciplines in healthcare, students indicate a lack of instructors with requisite skills in EBP especially in the clinic [12-14]. Incorporating education on the importance, use, and execution of RCTs, systematic reviews, and meta-analyses may improve knowledge of these research designs. Similarly, professional training on the use of EBP may improve the knowledge, skills, and beliefs of clinical physiotherapists and encourage a transfer of knowledge to physiotherapy trainees [15].

The overall attitude of respondents toward the use of RCTs, systematic reviews, and meta-analyses was good. Similarly, studies exploring the attitudes of clinicians toward EBM have reported positive attitudes from responding clinicians [9,16]. Exposure to systematic reviews and meta-analyses during graduate and postgraduate training and a good level of knowledge were significant factors independently associated with the attitude of physiotherapists toward the use of RCTs, systematic reviews, and meta-analyses. However, none of these factors predicted a positive attitude toward the use of RCTs, systematic reviews, and meta-analyses in our survey. Historically, physiotherapists display low regard for literature and research with respect to clinical practice, citing difficulty in reading journals and recognizing thought leaders [17]. However, new-generation physiotherapists display less aversion toward research [18-20]. Quality research can be quite rigorous and daunting while requiring funds and mentorship. This rigor and inaccessibility to funds can discourage the already busy clinician [20]; however, it remains essential to evidence-based clinical practice. An intrinsic motivation [19] and an enabling environment are required to improve attitudes toward research among practicing physiotherapists. Further studies to determine the predictors of a positive attitude to RCTs, systematic reviews, and meta-analyses are essential. Unveiling these factors may inspire policy implementation for their availability.

Our analysis revealed that the self-rated and overall level of knowledge of RCTs and meta-analyses were significant predictors of the professional use of RCTs, systematic reviews, and meta-analyses by Nigerian physiotherapists. Physiotherapists with advanced training in climes where research is prioritized have positive attitude, and that may influence an increased use of research and literature for evidence-based practice [18]. Further, improved confidence consequent of prior training in the use of literature for EBP may also significantly increase the use of RCTs, systematic reviews, and meta-analyses among physiotherapists [15].

Our study had some limitations. Information on the respondents’ geopolitical zones of practice was not collected. Furthermore, we used an electronic survey for members of the major professional associations in Nigeria; the participants who may not have access to the internet at the time of the survey and those who were yet to be added to these electronic platforms may have been unintentionally excluded, posing the risk of a selection bias. In addition to the small sample size, this may limit the generalizability of our results to the Nigerian national population of physiotherapists. However, these professional electronic platforms are representative of physiotherapists in every part of the nation. Additionally, we ensured a repetitive and consistent broadcast of the electronic survey for three months, providing the opportunity to reach as many physiotherapists as possible. Exposure to systematic reviews and meta-analyses during graduate and postgraduate training and the presence of a medical library in the workplace were the major predictors of the good knowledge and use of RCTs, systematic reviews, and meta-analyses among Nigerian physiotherapists. Additionally, the self-rated and overall level of knowledge were significant predictors of the professional use of RCTs, systematic reviews, and meta-analyses among physiotherapists in Nigeria. Furthermore, the majority of responding physiotherapists believed that their methodological knowledge should be improved.

## Conclusions

In conclusion, graduate and professional training should incorporate and prioritize training on the importance and use of RCTs, systematic reviews, and meta-analyses to inform clinical decisions and practice, while capable workplaces should set up medical libraries to ease access to and the use of RCTs, systematic reviews, and meta-analyses.

Furthermore, these predictors are dynamic and may change over time. Thus, future studies on identifying the factors that predict the attitudes of physiotherapists toward using research for EBP should be prioritized.
